# A device for difficult oronasal exchange of nasobiliary catheter

**DOI:** 10.1093/gastro/goag089

**Published:** 2026-08-20

**Authors:** Xiaoqing Zhang, Mingwen Guo

**Affiliations:** Department of Gastroenterology, The First College of Clinical Medical Science, China Three Gorges University, Yichang, Hubei 443003, P. R. China; Department of Gastroenterology, Qionglai Medical Center Hospital, Qionglai, Sichuan 611530, P. R. China

## Introduction

Endoscopic retrograde cholangiopancreatography (ERCP), which has been used for nearly 60 years, is essential in treating conditions like choledocholithiasis and biliary strictures [1]. Nasobiliary drainage catheter placement, a frequent ERCP procedure, is crucial for managing acute cholangitis and preventing post-extraction infections [2, 3]. After placement, the catheter must be moved from the mouth to the nose for easier postoperative care.

Conventionally, this procedure relies solely on a guidewire and rubber tube, which can be challenging in patients with endotracheal intubation or narrow pharyngeal cavities. Recently, modified techniques have been proposed to improve endoscopic nasobiliary drainage tube (ENBD) repositioning. Li *et al.* [4] developed an adjustable snare-assisted method, and Liu *et al.* [5] reported a syringe tube combined guidewire technique. Although these approaches improved procedural efficiency, both still rely on relatively straight instruments advancing through the oral cavity and generally require downward pressure on the tongue to reach the posterior pharyngeal wall. As a result, oral and pharyngeal mucosal irritation may still occur, potentially increasing patient discomfort and provoking gagging, nausea, or vomiting.

To address this issue, we developed a simple oronasal exchange device based on a modified oropharyngeal airway. Unlike straight instruments, the oropharyngeal airway conforms more closely to the natural curvature of the human oropharyngeal anatomy, allowing the guidewire to reach the posterior pharyngeal wall more easily while potentially reducing mechanical compression and mucosal injury to the oral cavity and pharynx.

## Case report

A 79-year-old female patient was admitted to our department with choledocholithiasis complicated by cholangitis and scheduled for ERCP stone extraction. She had previously undergone two open common bile duct explorations for choledocholithiasis and had a medical history of hypertension and coronary heart disease. The patient underwent ERCP under general anesthesia with tracheal intubation. Following ERCP and stone removal, a nasobiliary drainage catheter was placed.

**Figure 1:**
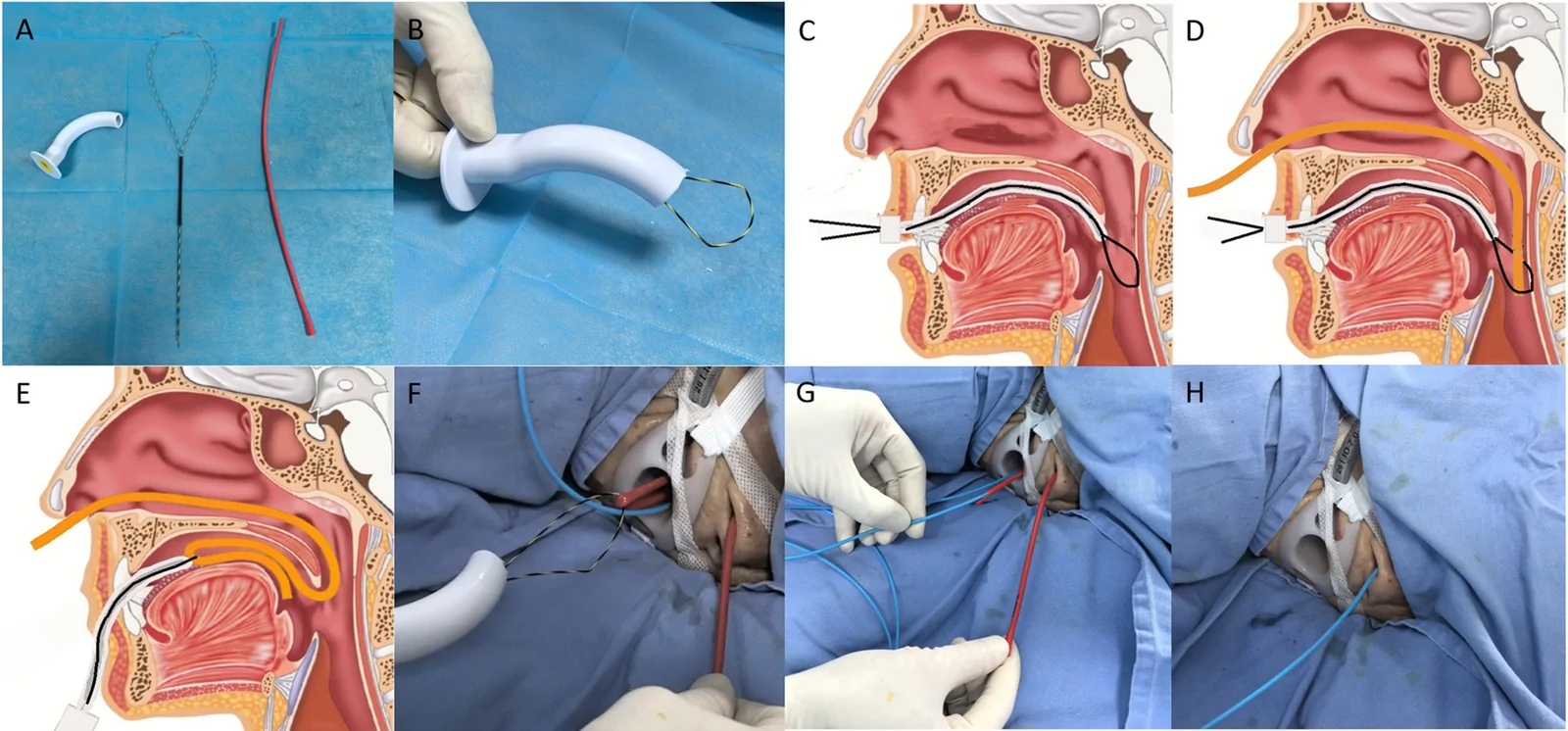
An oronasal exchange device for nasobiliary drainage catheters and key procedural steps. (A) Components of the oronasal exchange device, including the trimmed oropharyngeal airway, 0.035-inch zebra guidewire, and 12 Fr red rubber urethral catheter. (B) Preloaded guidewire shaped into a trapezoidal configuration (3 cm in width, 2 cm in length) at the distal end of the oropharyngeal catheter. (C) Schematic diagram: The modified oropharyngeal airway, preloaded with a guidewire, is inserted transorally into the pharynx, with the guidewire against the posterior wall to capture the rubber catheter. (D) Schematic diagram: Insert the soft rubber catheter transnasally to about 15 cm, ensuring the tip passes the guidewire loop. (E) Schematic diagram: The guidewire captures the rubber tube inserted transnasally as the device is gradually removed from the mouth. (F) The rubber tube passes from the nasal cavity into the oral cavity after device removal. (G) Connection of the nasobiliary catheter distal end into the side hole at the tip of the rubber tube. (H) After oronasal exchange, the nasobiliary catheter exits through the nose.

For the exchange procedure, we used an oropharyngeal catheter, guidewire, and soft rubber tube (Fig. 1). The oropharyngeal catheter was a conventional anesthetic oropharyngeal airway (Weigao, Shandong, China), with its distal end trimmed to an approximate length of 10 cm (Video S1) to ensure optimal positioning close to the posterior pharyngeal wall. The guidewire used was a 0.035-inch zebra guidewire (Micro-Tech, Nanjing, China), routinely employed in ERCP procedures. The soft rubber tube was a 12 Fr/38 cm red rubber urethral catheter (Jiangyang, Yangzhou, China). Prior to the procedure, all devices were adequately lubricated with oxybuprocaine gel (Jinling Pharmaceutical, China), and nasal lubrication and anesthesia were also performed using oxybuprocaine gel to minimize mucosal trauma and facilitate device passage (Video S1). Gentle manipulation was maintained throughout the entire procedure to avoid pharyngeal or nasal mucosal injury. This technique is contraindicated in patients with coagulopathy, severe nasal obstruction, or known nasopharyngeal anatomical abnormalities. The guidewire was preloaded into the oropharyngeal catheter and shaped to form a trapezoidal configuration approximately 3 cm in width and 2 cm in length at the distal end of the device (Fig. 1), which enabled the guidewire to better adhere to the posterior pharyngeal wall. Under direct visualization, the device was slowly inserted over the tongue and past the nasobiliary catheter and endotracheal tube until it reached the posterior pharyngeal wall (Fig. 1, Video S1). After inserting the soft rubber tube 15 cm through the nasal cavity (Fig. 1), the device was slowly withdrawn from the oral cavity, and the guidewire successfully captured the rubber tube (Fig. 1). Upon removal of the device, the rubber tube passed from the nasal cavity into the oral cavity (Fig. 1). The distal end of the nasobiliary catheter was inserted into the side hole at the tip of the rubber tube (Fig. 1). Finally, the rubber tube on the nasal side was gently pulled to withdraw the nasobiliary catheter through the nasal cavity, completing the oronasal exchange (Fig. 1, Video S1).

## Discussion

Our technique differs from previously reported ENBD repositioning methods in that it uses a modified anesthetic oropharyngeal airway, adapted from a routinely used airway device, as the guiding channel for oral-to-nasal exchange. Unlike relatively straight devices, the oropharyngeal airway follows the natural curvature of the oral cavity and pharynx and may therefore facilitate easier access to the posterior pharyngeal wall with less tongue compression. By contrast, previously reported techniques generally rely on relatively straight instruments, which may increase oral and pharyngeal stimulation when advanced toward the posterior pharyngeal wall [4, 5]. Our anatomy-adapted design may reduce mucosal friction and procedure-related irritation while remaining simple and readily applicable in routine practice. However, this is a single-case report, and these potential advantages require validation in larger comparative studies.

## Supplementary material

Supplementary material is available at *Gastroenterology Report* online.

## Authors’ contributions

X.Z. conceived and designed the project. X.Z. collected the data and drafted the manuscript. M.G. revised the manuscript critically for important intellectual content. All authors read and approved the final manuscript.

## Supplementary Material

goag089_Supplementary_Data
